# Graphene-Coated Nanowire Waveguides and Their Applications

**DOI:** 10.3390/nano10020229

**Published:** 2020-01-28

**Authors:** Da Teng, Kai Wang, Zhe Li

**Affiliations:** 1 School of Physics and Electronic Engineering, Zhengzhou Normal University, Zhengzhou 450044, China; 2 Key Laboratory of Infrared Imaging Materials and Detectors, Shanghai Institute of Technical Physics, Chinese Academy of Sciences, Shanghai 200083, China; 3 College of Sciences, Shanghai University, Shanghai 200444, China; lizhecn@shu.edu.cn

**Keywords:** graphene-coated nanowires, graphene plasmons, mid-infrared waves, waveguide

## Abstract

In recent years, graphene-coated nanowires (GCNWs) have attracted considerable research interest due to the unprecedented optical properties of graphene in terahertz (THz) and mid-infrared bands. Graphene plasmons in GCNWs have become an attractive platform for nanoscale applications in subwavelength waveguides, polarizers, modulators, nonlinear devices, etc. Here, we provide a comprehensive overview of the surface conductivity of graphene, GCNW-based plasmon waveguides, and applications of GCNWs in optical devices, nonlinear optics, and other intriguing fields. In terms of nonlinear optical properties, the focus is on saturable absorption. We also discuss some limitations of the GCNWs. It is believed that the research of GCNWs in the field of nanophotonics will continue to deepen, thus laying a solid foundation for its practical application.

## 1. Introduction

The manipulation of light-matter interaction at subwavelength scale using surface plasmons (SPs) [1,2,3], which could confine the electromagnetic fields into regions far below the diffraction limit [4], has been widely exploited due to various applications [5,6,7,8]. However, the inherent shortages of the metallic structures hinder their application in some plasmonic devices.

Graphene [9], a two-dimensional (2D) carbon material, was proved to be an alternate material that could excite SPs in the mid and far-infrared bands owing to its unprecedented properties such as the tunability, extremely strong modal field confinement, and huge field enhancement [10,11,12]. Therefore, lots of graphene-based novel optical devices, including waveguides [13,14,15,16,17,18,19], modulators [20,21], switches [22,23,24], polarizers [25,26], sensors [27,28], antenna [29], etc., have been developed, and the research of graphene plasmonics has promoted the development of nanophotonics. Particularly, the GCNWs [30,31,32,33,34,35] have aroused lots of research interest, and the wide applications of GCNWs in photonic devices have become one of the research hot spots in recent years. It is worth mentioning that although there already exists a review of graphene plasmonic waveguides [36], not enough emphasis is laid on the GCNWs and their applications.

Here, we review the latest research status of photonic devices based on GCNWs with a particular emphasis on various kinds of GCNW waveguides and their applications in photonic devices, nonlinear devices, lasers, and other new interesting fields. In order to get a better understanding of the condition of the graphene supporting surface plasmon modes, we first look back at the surface conductivity of graphene. We hope that the GCNWs will play a key role in future development of photonic devices.

## 2. Surface Conductivity of Graphene

The optical properties of graphene are described by its complex surface conductivity *σ*_g_ = *σ*_r_ + *jσ*_i_, where *σ*_r_ and *σ*_i_ represent the real and imaginary parts, respectively. For a graphene layer with thickness of *d*, the equivalent complex permittivity is given as [12,37]:

(1) $$\varepsilon_{g}=\varepsilon_{0}-\frac{\sigma_{i}}{\omega d}+\frac{\sigma_{r}}{\omega d}j$$

where *ε*_0_ is the vacuum permittivity and *ω* is the angular frequency of the light. The real (imaginary) part of *ε*_g_ is related to the imaginary (real) part of *σ*_g_. For a sufficiently small *d*, the real part could be approximately written as

(2) Re(εg)≈−σi/(ωd)

It can be seen that the real part of equivalent permittivity for the *d*-thick graphene layer can be positive or negative decided by the sign of the imaginary part of the graphene conductivity. When *σ*_i_ > 0, i.e., Re(*ε*_g_) < 0, the graphene layer shows “metallic“ properties, and could support a transverse-magnetic (TM) surface plasmon mode [1].

Further, the relative equivalent complex permittivity of graphene can be calculated by using *ε*_g_ = 1 + *jσ*_g_/(*ε*_0_*ωd*) [12], where *d* = 0.335 nm is the thickness of monolayer graphene [38,39]. Within the random-phase approximation, the dynamic optical response of graphene can be derived from the Kubo’s formula consisting of the interband and intraband contributions in the infrared ranges, that is *σ*_g_ = *σ*_intra_ + *σ*_inter_, and the surface conductivity of graphene is given as [40]:

(3) $$\sigma_{g}=\sigma_{\mathrm{intra}}+\sigma_{\mathrm{inter}}$$

(4) $$\sigma_{\mathrm{intra}}=\frac{2je^{2}k_{B}T}{\pi \hbar^{2}(\omega +j/\tau )}\ln[2\cosh(\frac{\mu_{c}}{2k_{B}T})]$$

(5) $$\sigma_{\mathrm{inter}}=\frac{e^{2}}{4\hbar }[\frac{1}{2}+\frac{1}{\pi }\arctan(\frac{\hbar \omega -2\mu_{c}}{2k_{B}T})-\frac{j}{2\pi }\ln\frac{{\left(\hbar \omega +2\mu_{c}\right)}^{2}}{{\left(\hbar \omega -2\mu_{c}\right)}^{2}+{\left(2k_{B}T\right)}^{2}}]$$

where *τ* is the electron relaxation time, *T* = 300 K is the temperature, *μ*_c_ is the chemical potential of the graphene, *ℏ* is the reduced plank constant, *k_B_* is the Boltzmann’s constant, and *e* is the charge of the electron. In the calculation, the graphene could be either treated as a thin layer or surface current. For convenience, graphene is usually modelled as an electric field-induced surface current **J** = *σ*_g_**E** on the surface of nanowire.

The tunability of the graphene is of great importance for practical applications in photonic devices. As we have shown before, the equivalent permittivity of graphene can be positive or negative depending on the sign of the imaginary part of the graphene conductivity. Hence, the imaginary part of the graphene conductivity *σ*_i_ determines the propagation of transverse-electric (TE) or TM modes. Clearly, when *σ*_i_ > 0 (Re(*ε*_g_) < 0), the graphene layer could support a TM SP mode. On the contrary, when *σ*_i_ < 0, the graphene layer could support a TE mode.

**Figure 1:**
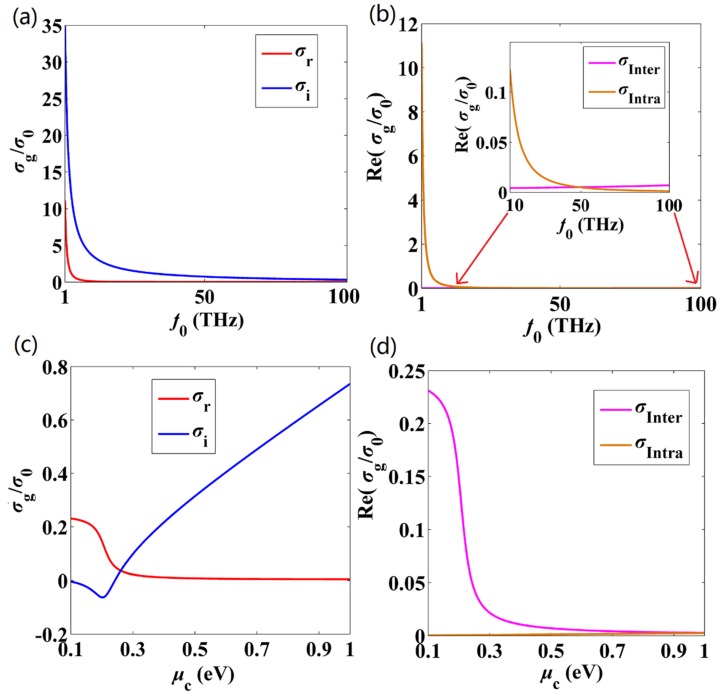
The graphene conductivity *σ*_g_, normalized by *σ*_0_ = *e^2^/ℏ*, as functions of the frequency and chemical potential, calculated from Equation (3). (**a**) Real and imaginary parts of *σ*_g_ with respect to frequency. (**b**) Interband and intraband contributions of the real parts of the graphene conductivity with respect to frequency. (**c**) Real and imaginary parts of *σ*_g_ with respect to *μ*_c_. (**d**) Interband and intraband contributions of the real parts of the graphene conductivity with respect to *μ*_c_. The chemical potential *μ*_c_ is 0.5 eV for (**a**,**b**), and the frequency is 20 THz for (**c**,**d**). Other parameters are *τ* = 0.5 ps and *T* = 300 K.

Figure 1 depicts the surface conductivity of the graphene as functions of frequency and chemical potential. Here, we focus on the terahertz and mid-infrared bands, thus *f*_0_ ranges from 1 THz~100 THz. The graphene conductivity values are all normalized by *σ*_0_ = *e^2^/ℏ*. One can see from Figure 1a that for the frequencies considered here, *σ*_i_ is always above 0, and TM modes can be propagated. The interband and intraband contributions of the real parts of the graphene conductivity Re(*σ*_g_) are shown in Figure 1b. It indicates that the intraband contribution dominates in the low frequency range, that is *σ*_g_ ≈ *σ*_inter_. While at higher frequency range (*f*_0_ > 48 THz), the interband contribution takes over. Therefore, the intraband (interband) contribution is responsible for loss at low (high) frequencies. In Figure 1c, we plot the real and imaginary parts of *σ*_g_ with respect to *μ*_c_. It is found that at very small *μ*_c_, *σ*_r_ is higher than *σ*_i_. Note that by increasing *μ*_c_, *σ*_r_ decreases to nearly zero, which indicates that a significant reduction in loss can be achieved by using high chemical potential values. For *μ*_c_ > 0.24 eV, *σ*_i_ > 0, and TM SP modes can be supported in the considered range. The interband and intraband contributions of the real parts of the graphene conductivity with respect to *μ*_c_ are shown in Figure 1d. It shows that the interband transition drastically reduces as *μ*_c_ increases, and the intraband contribution dominates at higher *μ*_c_. Also, intraband and interband contributions are at the same level, which suggests one can reduce the modal loss easily by enhancing *μ*_c_.

## 3. GCNW Waveguides

**Figure 2:**
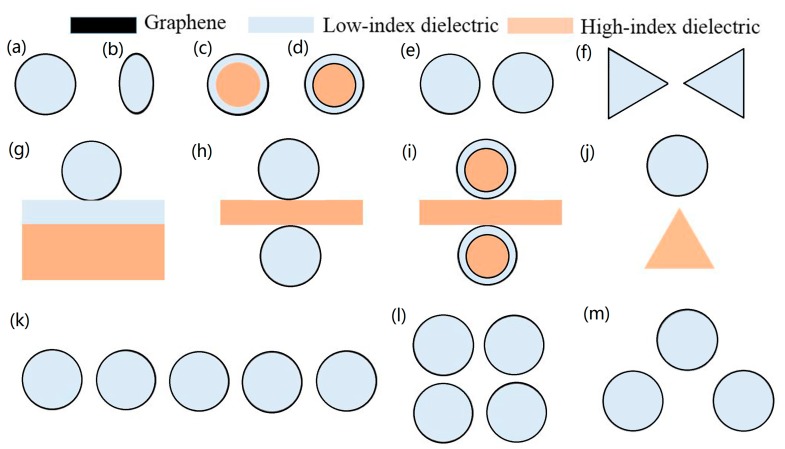
Various graphene-coated nanowire (GCNW)-based waveguides. (**a**) Circular and (**b**) Elliptical GCNWs. (**c**) Coaxial-like and (**d**) Long-range coaxial-like GCNWs. (**e**) Circular and (**f**) Bow-tie GCNW pairs. (**g**) Traditional and (**h**) symmetric GCNW-based hybrid waveguide. (**i**) Modified symmetric and (**j**) Triangle dielectric integrated GCNW-based hybrid waveguides. (**k**) 1D and (**l**) 2D arrays of GCNWs. (**m**) GCNW trimer.

In this section, we review the GCNW waveguides, and Figure 2 gives the cross-sections of various GCNW-based structures. We classify these structures into several groups: (a) and (b) for graphene-coated single circular/elliptical nanowire, (c) and (d) for coaxial-like GCNWs, (e) and (f) for GCNW pairs, (g–j) for GCNW-based hybrid waveguides, and (k–m) for GCNW 1D/2D arrays and trimer. For convenience, we have also listed the main parameters below to evaluate the waveguiding performances.

Usually, propagation length (*L*_P_), normalized mode area (*A*_nor_), and figure of merit (FoM) are employed to quantitatively illustrate the waveguiding performances. The propagation length *L*_P_ is defined as the amplitude of the field decays to 1/*e* of its initial value and calculated by *L*_P_ = *λ*_0_/[2πIm(*N*_eff_)], where *λ*_0_ is the wavelength in vacuum and Im(*N*_eff_) denotes the imaginary part of the effective mode index. The effective mode size *A*_eff_ is evaluated by the ratio of the total mode energy and the maximum electromagnetic energy density [41]:

(6) $$A_{\mathrm{eff}}=\iint W(r)d^{2}r/\max[W(r)],$$

where the electromagnetic energy density *W*(r) is given by

(7) $$W\left(r\right)=\frac{1}{2}\left\{\frac{d[\varepsilon \left(r\right)\omega ]}{d\omega }\left|E\left(r\right){|}^{2}+\mu_{0}\right|H\left(r\right){|}^{2}\right\}.$$

In Equation (7), E(r) and H(r) denote the electric and magnetic fields, respectively. *ε*(r) is the electric permittivity and *μ*_0_ is the magnetic permeability in air. The normalized mode size is defined as *A*_nor_ = *A*_eff_/*A*_0_ with $A_{0}=\lambda_{0}^{2}/4$ being the diffraction-limited mode size in air. Figure of merit (FoM) [42] is defined as the ratio of the mode propagation length to the diameter of the effective mode size, which is $\mathrm{FoM}=L_{p}/\sqrt{A_{\mathrm{eff}}/\pi }$.

### 3.1. Graphene-Coated Single Nanowires

#### 3.1.1. GCNWs

As far as we know, the study of the graphene-coated single nanowire (see Figure 2a) began with considering the photonic modes in THz region, where the graphene-coated semiconductor cylinder served as a photonic crystal [43]. Later, Zhao et al. [44] numerically studied the surface plasmon whispering gallery mode properties of the graphene-coated InGaAs nanowire cavity. A high cavity quality factor of 235 was obtained for a 5 nm radius cavity, along with a mode area (see Equation (6)) as small as 3.75 × 10^−5^ $\lambda_{0}^{2}$ (when normalized by $A_{0}=\lambda_{0}^{2}/4$, the normalized mode area is 1.5 × 10^−4^) at *λ*_0_ = 1.55 μm.

**Figure 3:**
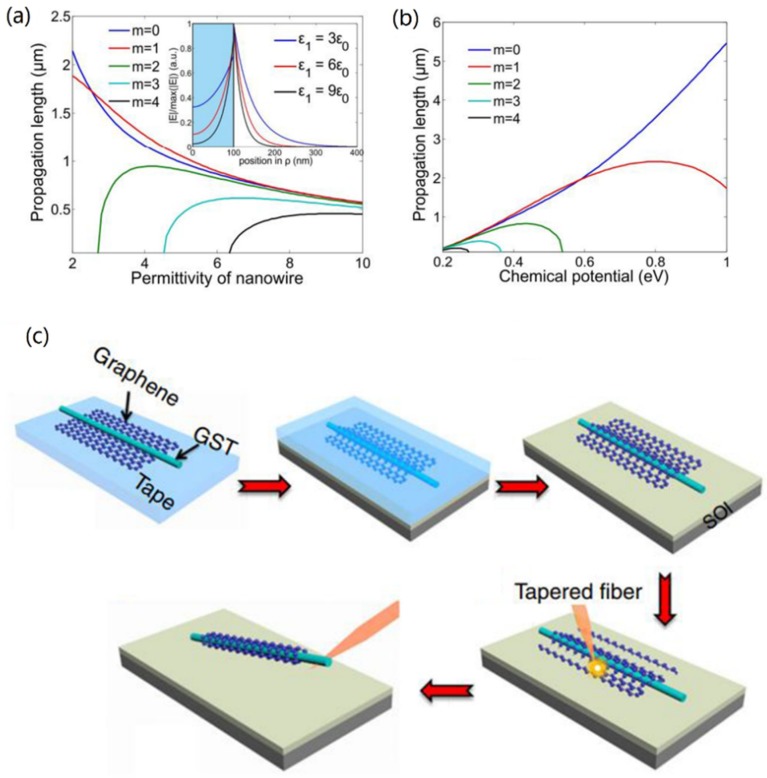
Propagation length as functions of nanowire permittivity (**a**) and chemical potential (**b**) at 30 THz with *R* = 100 nm. Reprinted with permission from reference [30]. Copyright Optical Society of America, 2014. (**c**) Schematic illustration of the GCNW fabrication process. Reprinted with permission from reference [46]. Copyright Springer Nature, 2018.

Important progress occurred in 2014. The eigen equation for the SP mode in the GCNW was achieved for the first time in THz [45] and mid-infrared bands [30]. It was found that the fundamental TM surface plasmon mode TM_0_ was cut-off free. In the work, Gao et al. [30] showed that with increasing nanowire permittivity, effective mode index almost linearly increased, which indicated a stronger localization of graphene plasmon mode, while larger modal loss (see Figure 3a). Also, high-order modes no longer existed at a small enough value of nanowire permittivity. A propagation length of 5.44 μm (see Figure 3b) and a normalized mode area of 1.35 × 10^−3^ could be obtained at 30 THz with a radius of 100 nm and chemical potential fixed at 1 eV. Meanwhile, Gao et al. [31] presented an analytic modal cut-off wavelength formula, which could be applied to calculate the cut-off wavelength of each order mode easily and accurately. Results also indicate that the higher-order modes would be cut-off at certain chemical potential values, which means the cut-off frequencies of these modes depend on the inductive behavior provided by the graphene layer.

Apart from the theoretical analysis of GCNWs, several research teams reported [46,47,48] the fabrication of freestanding GCNWs for optical waveguiding, and Figure 3c is the schematic illustration of the GCNW fabrication process [46]. Also, He et al. [48] investigated the graphene-covered microfiber, and both theoretical and experimental results indicated that the proposed device can be used as a polarization dependent saturable absorber as well as an optical polarizer. Thus, they showed the dielectric nanowire can be easily coated by a graphene layer due to the van der Waals force.

**Figure 4:**
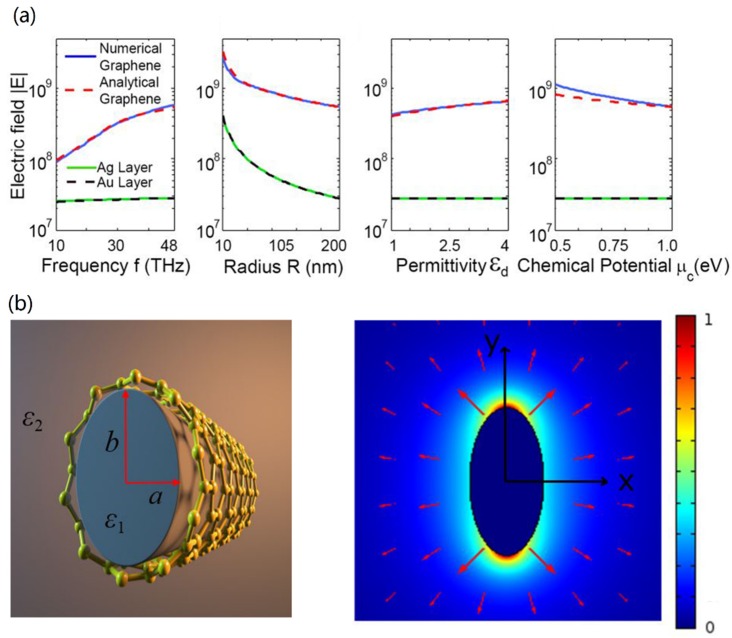
(**a**) The electric fields |E| of the graphene-coated and metal-coated nanowire waveguides as functions of frequency, nanowire radius *R*, nanowire permittivity, and chemical potential. Reprinted with permission from reference [49]. Copyright Optical Society of America, 2014. (**b**) Graphene-coated elliptical nanowire and normalized electric field distribution [54].

Owing to the strong optical field enhancement of graphene plasmons, Zhu et al. [49] proposed a graphene-coated tapered nanowire probe to achieve strong field enhancement in the mid-infrared frequencies. Based on the adiabatic approximation |d[Re(*β*)^−1^]/d*z*| << 1 [50], they analytically investigated the field enhancement along the tapered region, and results were consistent with the rigorous numerical simulations. Finally, the GCNW probe could achieve an order of magnitude larger field enhancement than the metal-coated probes, shown in Figure 4a. Later, they showed that a field enhancement as high as 24 could be achieved [51].

In 2016, Davoyan et al. [52] provided a performance comparison between the GCNW structure and some other typical THz waveguides. Results showed that the GCNW outperformed its metallic analog in modal field confinement, since surface plasmon modes in subwavelength metallic wire (∼*λ*_0_/50) radius were weakly confined at THz band. Later, Huang and Cong et al. [34,53] proposed the GCNWs with a drop-shaped cross-section for guiding THz plasmons, and an extremely long propagation length (1 mm) with a very small focal spot with full width at half maximum (FWHM) about 10 nm could be achieved, which resulted from the distinctive mode field distribution caused by both the top and bottom arcs of the waveguide. In 2019, Teng et al. [54] showed that graphene-coated elliptical nanowires (see Figure 4b) could be used for THz waveguiding, and a propagation length over 200 µm as well as a normalized mode area of approximately 10^−4^~10^−3^ could be obtained at 3 THz. Increasing long-short axis ratio could simultaneously achieve both long propagation length and very small FWHM of the focal spots. For *b*/*a* = 10, a pair of focal spots about 40 nm could be obtained. In mid-infrared band, the elliptical GCNW [55] was investigated in the elliptical cylinder coordinate system, and the dispersion equation was obtained by using the separation variable method with the Mathieu functions. A propagation length around 2 μm could be obtained when *a* = 110 nm, *b* = 80 nm, *μ*_c_ = 0.72 eV, and *λ*_0_ = 7 μm. The long and short axes of the elliptical nanowire have a significant influence on the mode properties. A few months before, Wu et al. [56] studied the dispersion equation of a GCNW, and found there was another branch of guided modes, called photonic-like modes. The propagation distances of these photonic-like modes could be five orders of magnitude longer than those of the SP modes.

It is worth mentioning that localized surface plasmon mode [57] and TE plasmon mode [58] in GCNWs have also been investigated. The report showed that the negativity of graphene conductivity’s imaginary part was not a sufficient condition, and the GCNW supported TE plasmon mode when the core radius of waveguide was larger than the critical value. Results also indicated that the critical radius depended on the frequency and the index-contrast between the inside and outside materials of the waveguide.

#### 3.1.2. Coaxial-like GCNWs

In 2016, Liu et al. [35] proposed another kind of GCNW waveguides, and we call them coaxial-like GCNW, shown in Figure 2c. The coaxial-like GCNW is composed of a silicon nanowire core surrounded by a silica layer and then a graphene layer, and it is also similar to the cylindrical hybrid plasmonic waveguide, which is composed of a low-index dielectric layer sandwiched between a high-index dielectric layer and a graphene layer (or metal). Based on analytical study and numerical simulation, an ultra-small normalized mode area about ~10^−5^ and a large propagation length about 8 μm can be achieved at the wavelength of 7 μm, which outperformed the graphene-coated single nanowires. Then, Liu et al. [59] proposed a coaxial-like GCNW long-range waveguide (see Figure 2d), in which an extra graphene layer on the silicon nanowire core was added. The symmetric coupling and anti-symmetric coupling mode originated from the constructive interference and destructive interference of the two GCNWs were intensively investigated. For the fundamental long-range (*L*_0_) mode, when *λ*_0_ = 10 µm, *μ*_c_ = 0.6 eV, *R* = 24 nm, and *t* = 12 nm, *L*_P_ is about 9 µm, while the normalized mode area is still ~10^−5^. Further, the propagation length could be increased with increasing *μ*_c_.

Recently, Zhao et al. [60] theoretically investigated the plasmon modes in a circular cylindrical double-layer graphene structure, which was similar to coaxial-like GCNW long-range waveguide. By solving the Maxwell equations together with boundary conditions, they obtained the electromagnetic fields in each region and then the dispersion equation. Particularly, results showed that the trade-off between mode confinement and propagation loss was broken at large inner graphene layer to outer graphene layer distances. As a consequence, both strong mode confinement and longer propagation length can be achieved. Also, the modal property of an elliptical coaxial-like GCNW [61] was analyzed by using the separation of variables method. The surface conductivity of graphene was modulated by employing a DC bias, which was highly important to study the tunable properties of the graphene-based plasmonic devices. Serrano et al. [62] investigated the propagation of surface plasmon modes along three kinds of electrically and magnetically biased GCNWs in THz band, and designed a plasmonic dipole antenna.

### 3.2. GCNW Pairs

The GCNWs have shown excellent optical performances, such as low propagation loss and fundamental mode cut-off free. However, the TM_0_ mode with a radially polarized electric field (with field components of E_r_, H_φ_, E_z_) in a GCNW remains weakly localized, since the field exponentially decays away from the interface. This problem is later addressed by using a GCNW pair, shown in Figure 2e,f, which is an analogy of metal two-wire waveguides [63,64].

**Figure 5:**
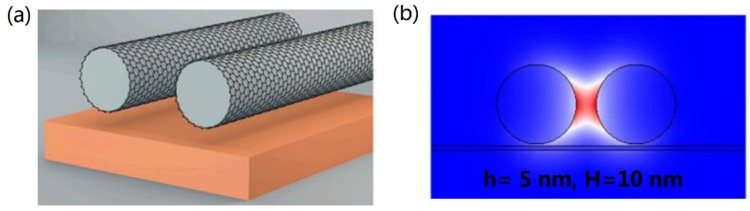
(**a**) Schematic of GCNW pair above a substrate, and (**b**) normalized electric field distribution of the fundamental graphene plasmon mode at 30 THz [69].

The study of GCNW pairs began with investigating the field enhancement and gradient force [65]. Then Zhai et al. [66] studied a GCNW pair with elliptical cross-section by using the finite element method, and found the modal properties can be adjusted finely by the elliptical semiminor axis. Peng et al. [67] analyzed the mode properties of an asymmetric GCNW pair waveguide by the multipole expansion method. These reports laid emphasis either on the field enhancement or effective mode index of the fundamental plasmon mode, while lacking a comprehensive evaluation of the waveguiding performances of GCNW pairs. In 2019, Teng et al. [33] fully investigated the waveguiding performance of the GCNW pairs in the mid-infrared range. Results indicated that the fundamental quasi-TM plasmon mode could achieve a propagation length about 9 μm, while the normalized mode area is only 10^−4^, which was one order of magnitude smaller compared with the GCNWs. Further investigations showed reducing nanowire permittivity could decrease the modal loss. Due to the circular geometry, the field was still weakly confined. Based on the bow-tie shaped metallic structures, a bow-tie type GCNW pair [68] was proposed, shown in Figure 2f. Benefiting from the sharp tip, the normalized mode area approached to an order of 10^−7^ magnitude, which was greatly reduced compared with other reports. Later, Teng et al. [69] theoretically showed that high-performance and low-loss transmission of graphene plasmons can be achieved by adding a silica substrate to the GCNW pairs, shown in Figure 5. Furthermore, the results showed that inserting a low index material layer between the nanowire and substrate could compensate for the loss accompanied by the substrate. Recently, Raad et al. [70] proposed a 3D graphene-coated nano-disk dimers to achieve multi-frequency near-field enhancement, which originated from the excitation of hybridized localized surface plasmons on top and bottom faces of the disks along with the mutual coupling from the adjacent particle.

The GCNW pairs show distinct advantage over the GCNWs. However, the trade-off between modal loss and field confinement still exists in the GCNW pairs, which hinders the practical applications of these configurations in integrated photonics. Despite the recent achievements, further reducing the modal field size while increasing (or maintaining) the transmission distance remains a huge challenge. On the other hand, in most previous studies, GCNWs and GCNW pairs are assumed to be surrounded by air, which means that the GCNWs are suspended without support. For applications in photonic integration circuits [71], a buffer or substrate is indispensable. Next, we will show how to address this obstacle.

### 3.3. GCNW-Based Hybrid Waveguides

To further enhance the optical performances of the GCNWs, it was proposed that when the GCNW was located adjacent to a high-index dielectric substrate, a substrate-supported GCNW with extreme small modal field can be realized. Meanwhile, a huge reduction of the modal propagation distance emerges due to the optical energy leakage into the substrate, which causes a great challenge for the implementation of long-range optical energy transmission.

Inspired by the metallic hybrid plasmon waveguide [41], which consists of a dielectric nanowire separated from a metal surface by a nanoscale dielectric gap. Hajati et al. [72] theoretically showed that high-performance and low-loss mid-infrared plasmon waveguiding could be achieved by inserting a thin low-index dielectric layer between a GCNW and a high-index substrate, shown in Figure 2g. It is well known that this hybrid mode originated from the coupling of graphene plasmon mode and the photonic mode inside the high-index substrate, which is similar to metal hybrid plasmon waveguide [41]. Therefore, the GCNW-based hybrid waveguides offer a better compromise between the loss and confinement than GCNWs. In 2017, Hajati et al. [73] proposed a symmetric GCNW-based hybrid waveguide, which comprised two vertically coupled GCNWs integrated with a thin high-index dielectric substrate, shown in Figure 2h. Through optimal design, a surface plasmon mode with high optical performance and low propagation loss can be achieved in the proposed structure. Results also showed a highly improved FoM with nearly two-fold electric field enhancement can be achieved compared with the plasmon mode in a GCNW over substrate. The modal propagation length could exceed 10 μm, and the normalized mode area is only 10^−5^ (see Equation (6)).

**Figure 6:**
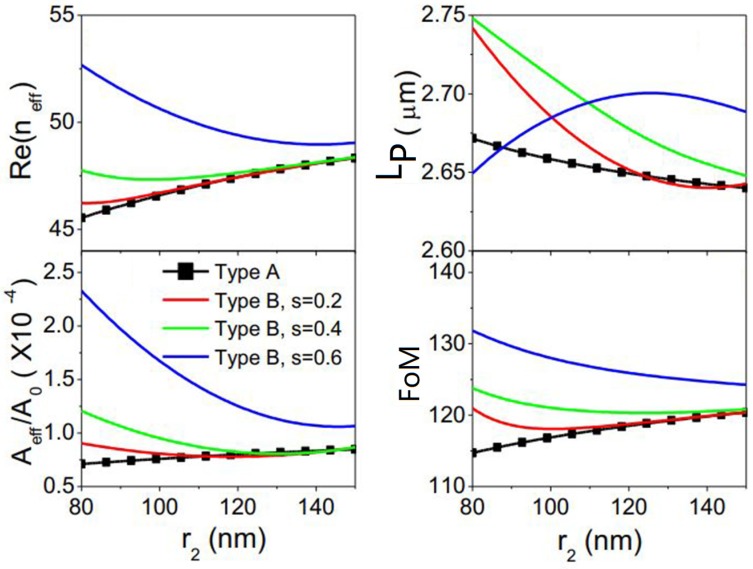
Comparison of the performance of two kinds of waveguides illustrated in Figure 2h,i. Reprinted with permission from reference [75]. Copyright Elsevier, 2019.

Later, Wu et al. [74] further reduced the normalized mode size down to only 10^−7^ by using a GCNW-based hybrid waveguide integrated with triangle wedge substrate and the low-index dielectric gap, shown in Figure 2j. At the same time, the graphene plasmon mode could propagate several micrometers. Also, a modified symmetric GCNW-based hybrid waveguide [75] consisting of two vertically coupled double-graphene-coated nanowires integrated with a thin high-index dielectric substrate was investigated (see Figure 2i). Results showed under certain conditions, the proposed waveguide (Figure 2i, Type B) has better mode performance (FoM) over the symmetric GCNW-based hybrid waveguide (Figure 2h, Type A), shown in Figure 6. A novel hybrid plasmonic waveguide [76] based on the graphene-coated V-groove and the GCNWs was also proposed.

In the literature, a lot of promising graphene-based hybrid waveguides [77,78,79,80,81,82,83,84,85,86] were analyzed in THz and mid-infrared bands. Here, we mainly focus on the GCNWs and GCNW-based hybrid waveguide, thus they will not be covered here.

### 3.4. GCNW Arrays

**Figure 7:**
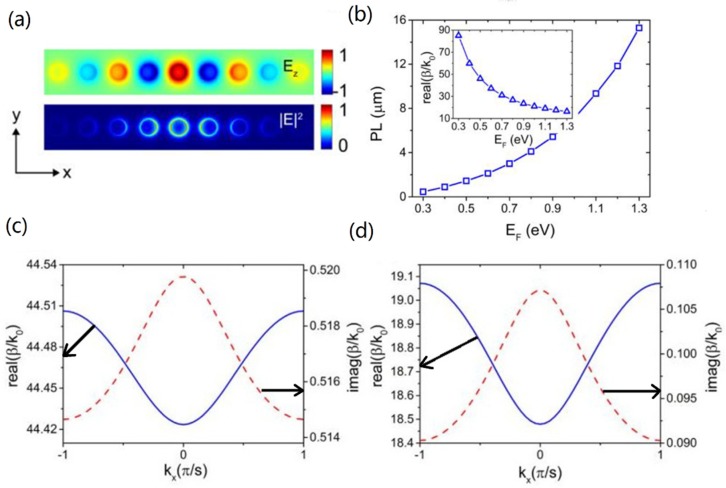
(**a**) Field distribution of a 1D GCNW array. (**b**) The soliton propagation length, insert for soliton mode index. Diffraction relation of the fundamental transmission band, *μ*_c_ = 0.5 eV for (**c**) and 1.1 eV for (**d**). *λ*_0_ = 10 μm, *a* = 100 nm, and *s* = 4*a*. Reprinted with permission from reference [88]. Copyright Optical Society of America, 2016.

Recently, graphene plasmonic solitons were predicted in graphene planar sheets and ribbons [87]. Also, researchers found that discrete plasmonic soliton modes could exist in 1D and 2D arrays of GCNWs [88], shown in Figure 2k,l. The waveguides coupling, discrete diffraction, as well as nonlinear modes were investigated by strictly solving the Maxwell’s equations. Results showed the soliton propagation length could over 10 μm, shown in Figure 7b. It can be seen that the real part of the diffraction curve minimized at the Brillouin zone center (*k*_x_ = 0) (see Figure 7c,d), implying a negative coupling as in other types of plasmonic waveguides. The increased band curvature at *μ*_c_ = 1.1 eV reflected the stronger discrete diffraction. Meanwhile, the imaginary part, which reflected the propagation loss of the Bloch modes, arrived at its minimum at the edge of the Brillouin zone (*k*_x_ = π/s), and significantly decreased as the coupling became stronger (at larger *μ*_c_). Later, Meng et al. [89] investigate the Bloch and topological edge plasmon modes in a 2D GCNW arrays. Due to the strong confinement of graphene plasmons, the modal wavelength of topological edge modes can be only *λ*_0_/20. Each gap possessed two degenerate topological edge modes. The imaginary parts of effective mode indices of the topological edge modes are 0.378 and 0.42, and the corresponding propagation distances are 2.53 μm and 2.27 μm, respectively.

**Figure 8:**
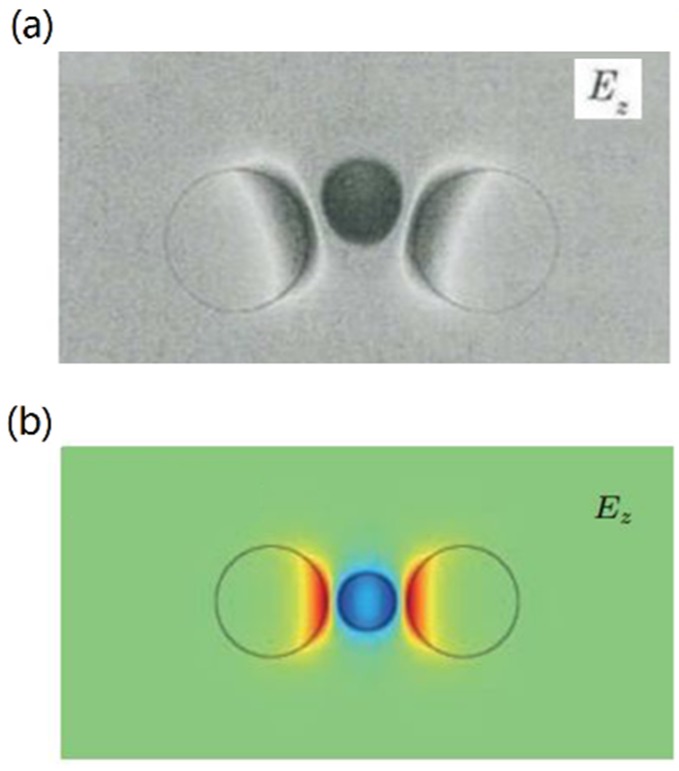
Field distributions (E_z_) of the lowest-order mode in non-coplanar (**a**) and coplanar (**b**) GCNW trimers. (**a**) Reprinted with permission from reference [90]. Copyright Chinese Laser Press, 2019. (**b**) Reprinted with permission from reference [91]. Copyright Chinese Physical Society, 2018.

Recently, the trimers of GCNWs (see Figure 2m) with a non-coplanar [90] and coplanar [91] axis were also proposed to guide mid-infrared and terahertz waves, and the lowest modal fields were shown in Figure 8a,b. These two structures were analyzed to obtain the effective mode index by using the multipole method. The first five modes were systematically investigated in terms of field distributions and propagation properties, and the analytical results were consistent with the simulations.

Apart from the study of the modal propagation properties, and the excitation of plasmon modes in the GCNWs was also significant. Usually, surface plasmon modes could be excited by using periodic structures such as metal gratings [92,93]. Several approaches [94,95,96,97,98] have been proposed to excite graphene plasmons in graphene planar structures. In 2016, Xia et al. [99] demonstrated an effective solution to excite localized surface plasmons on GCNW arrays, and the excited localized mode was compared with that of graphene nanoribbons. The excited resonance frequency showed an obviously blue shift, along with the narrowing of FWHM, which was due to low occupation ratio and smaller width of the incident light in rolled graphene, thus only the shorter wavevector can couple into the plasmon wave.

## 4. Applications of GCNWs

As indicated before, plasmon modes in GCNWs offer some important advantages, for example, tunability by changing the surface conductivity, extremely strong modal field localization, and huge field enhancement. These unprecedented properties make a good promotion of various applications in many fields. Here, we briefly review some of the major applications of GCNWs.

### 4.1. Photonic Devices

**Figure 9:**
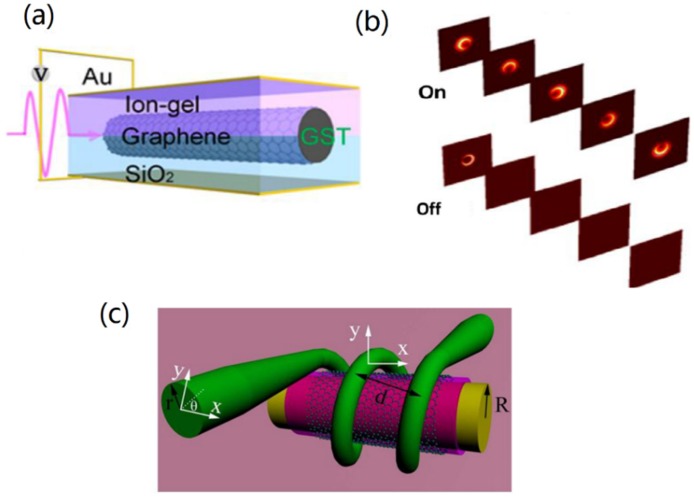
(**a**) Sketch of the graphene-coated GST225 nanowire and (**b**) switching “on/off“ chiral SP modes at *μ*_c_ = 0.6 eV. Reprinted with permission from reference [101]. Copyright American Chemical Society, 2018. (**c**) Schematic of a graphene-based nanowire in-line polarizer with a two-coil structure. Reprinted with permission from reference [105]. Copyright Optical Society of America, 2014.

The applications of GCNW-based tunable nanoscale devices are very important in photonics integration. Based on graphene cylindrical resonators, Asgari et al. [100] proposed a refractive index sensor, a power splitter, and a four-channel multi/demultiplexer. The proposed structure was composed of two or four graphene sheets as its input/output ports and a graphene cylindrical resonator. Cao et al. [46,101] proposed a chiroptical switch based on chiral plasmons in a graphene-coated Ge_2_Sb_2_Te_5_ (GST225) nanowire. Results showed that the chiral SPs propagating along the nanowire can be reversibly switched between “on“ (transparent) and “off“ (opaque) as transiting the state of GST225 core between amorphous and crystalline, shown in Figure 9a,b. And the short phase transition times of 2.4 ns and 8 ns can be obtained, resulting in a fast switching on/off. Gan et al. [102] proposed an all-fiber phase shifter based on graphene’s strong optical absorption and excellent thermal properties, and the proposed graphene-coated microfiber enabled all-optical switching with an extinction ratio of 20 dB and a rise (fall) time of 9.1 ms (3.2 ms).

Optical isolators [103] are crucial for nanoscale photonic applications. Pae et al. [104] demonstrated a graphene waveguide ring resonator, consisting of a GCNW and a graphene layer, allowed a nanoscale platform for a high-contrast optical isolator. The magnetically controlled graphene structure for nanoscale high-contrast optical isolator was due to plasmon resonance enhancement combined with resonator resonance enhancement.

Polarization behavior has a profound impact on the performance of optical devices. The control of polarization in the GCNW was reported in Reference [48]. It was reported that the saturated absorption could be achieved by using a graphene layer covered on the upper surface of the microfiber. Particularly, when the microfiber radius reduced to 1 μm, such graphene-microfiber hybrid waveguide can be utilized as TM polarizer, which implied that the graphene-coated microfiber could be used for optical communication. Subsequently, Kou et al. [105] demonstrated polarization manipulation by a GCNW. The ultra-long light–graphene interaction was implemented by a graphene-integrated helical microfiber device, shown in Figure 9c. The proposed device can operate as not only a broadband polarizer but also a high-Q (nearly 2 × 10^4^) single-polarization resonator. By employing a two-coil structure, an extinction ratio as high as ∼16 dB was obtained over a 450 nm bandwidth.

### 4.2. Ultrafast Optical Modulators

Electro-optic modulators are among the most important components for optical communication. For photonic integration, optical modulators with high modulation speed, small size, and large bandwidth are preferred.

**Figure 10:**
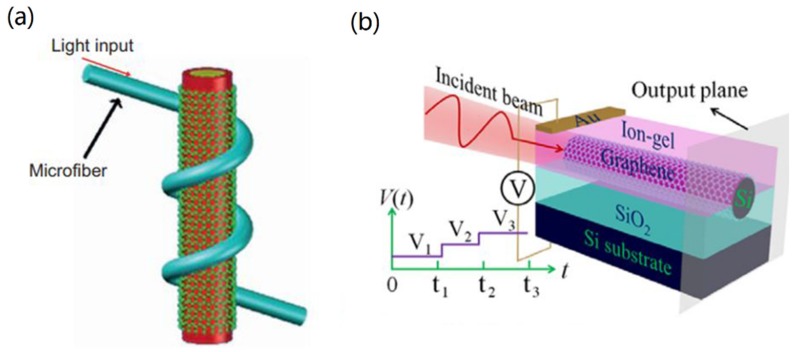
(**a**) A stereo graphene-coated–microfiber modulator. Reprinted with permission from reference [108]. Copyright Springer Nature, 2015. (**b**) Gate-programmable electro-optic addressing scheme. Reprinted with permission from reference [109]. Copyright American Chemical Society, 2016.

In 2011, Liu et al. [106] demonstrated a high-speed graphene-based optical modulator. By electrically tuning the Fermi level of a graphene film, a broad operation spectrum and very small device area of merely 25 μm^2^ were achieved. Later in 2014, Li et al. [107] reported that a graphene-coated microfiber all optical modulator could achieve a modulation depth of 38% and a response time of ∼2.2 ps. Also, an in-line, all-optical fiber modulator based on a stereo graphene-coated microfiber structure was demonstrated [108], shown in Figure 10a. The all-optical modulation mechanism was based on the Pauli blocking effect. Results showed a modulation depth of 7.5 dB and a modulation efficiency of 0.2 dB/mW could be achieved.

Recently, Liang et al. [109] derived the eigenmode equations of both tightly confined EH (Plasmon modes dominated by a longitudinal electric field) and TM SP modes supported by the GCNW and analytically and numerically studied their modal characteristics. Significantly, both the period of the swing beam and the chirality and period of the helix could be modulated by tuning the applied gate voltage on graphene. The proposed nanowire system, shown in Figure 10b, offered a way for nanoscale photonic devices at sub-10 nm scale.

### 4.3. Nonlinear Optics

Graphene plasmonic waveguides offer huge field intensity on the surface of graphene, which will enhance nonlinear effects significantly. We outline the main applications of GCNW in this field.

#### 4.3.1. Saturable Absorber in Fiber Lasers

In this subsection, we show that the GCNWs could work as a saturable absorber in fiber lasers. In 2012, He et al. [110] reported that based on the reduced graphene oxide (RGO) deposited on the surface of the microfiber by use of high-temperature heating, a direct generation of doublet ultra-wide-band pulses were observed by using of a passively mode-locked fiber laser. The strong interaction of the RGO with the evanescent field of the microfiber in the fiber laser system caused the saturable absorption effect. Also, results indicated the ultra-wide-band doublet pulses could be directly generated through the interaction between the dispersion and nonlinearity in the laser cavity. However, the modulation depth was only 5.75%. In 2014, the same team investigated a polarization-dependent saturable absorber [48] based on a graphene layer covered on the upper surface of the microfiber. Through reducing the radius of microfiber, the strong light–graphene interaction occurred via the evanescent field of the guided mode in the microfiber. When the radius of the microfiber is 0.8 μm, its polarization extinction ratio is ∼27 dB. When the radius of microfiber reached ∼3 μm, a polarization-dependent saturable absorber could be obtained with high thermal damage threshold of ∼975.82 MWcm^−2^ for p-polarization and ∼1233.2 MWcm^−2^ for s-polarization, and its polarization-dependent modulation depth varied from ∼10.25% to ∼12.85%. In the same year, Zhao et al. [111] reported on the generation of dual-wavelength rectangular pulses in a Yb-doped fiber laser by using a graphene-coated microfiber saturable absorber.

**Figure 11:**
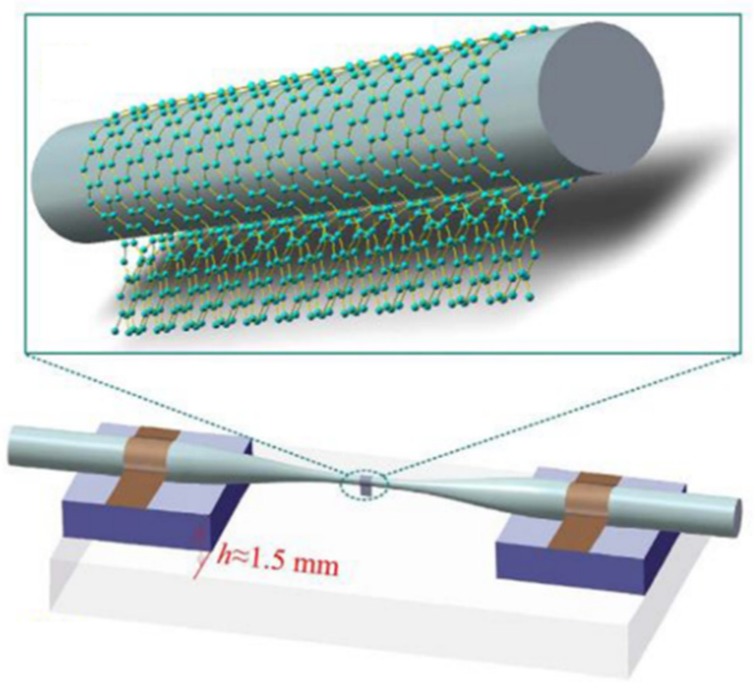
The GCNW saturable absorber. Reprinted with permission from reference [112]. Copyright Springer Nature, 2016.

Later, Liu et al. [112] exploited a graphene-coated microfiber saturable absorber (see Figure 11) in a mode-locked fiber laser for the generation of ultrafast pulses. The proposed all-surface technique can realize a higher efficiency of light–graphene interactions, and the GCNW-based saturable absorber could generate ultrafast optical pulses within 1.5 μm. Yao et al. [113] demonstrated a compact all-in-line graphene-based distributed feedback Bragg-grating fiber laser with narrow linewidth based on GCNWs. In 2018, Wang et al. [114] reported that using a graphene-coated microfiber saturable absorber, the generation and evolution of multiple operation states were proposed and demonstrated in passively mode-locked thulium-doped fiber laser. Recently, Li et al. [115] reported an active–passive Q-switched laser based on graphene-covered microfiber, which not only served as a passive saturable absorber in a single laser cavity, but also could be used as an all-optical modulation device.

#### 4.3.2. Optical Bistability

Optical bistability [116] is a rapidly expanding field of current research because of its potential application to all-optical logics and switching. However, in nonlinear plasmonics, the switching threshold of optical bistability is limited by the weak nonlinear responses from the conventional Kerr dielectric media. To enhance the nonlinear responses, GCNWs were employed to beat this limitation. Based on GCNWs, Li et al. [117] developed a nonlinear scattering model under the mean field approximation and studied the bistable scattering in a GCNW based on the semi-analytical solutions. It was found that the switching intensities of bistable scattering can be smaller than 1 MW/cm^2^ at the working frequency. Meanwhile, another team [118] studied the optical bistability of GCNWs with Kerr-type nonlinear response within the framework of both nonlinear full-wave scattering theory and nonlinear quasi-static theory. Typical optical bistable properties were observed, and results indicated when high electromagnetic field was applied, nonlinear full wave theory yielded a new bistable region, indicating the existence of an artificial tunable magnetic dipole. These results could offer guidance to application of optical bistability in the high-speed all-optical communication.

#### 4.3.3. Other Nonlinear Effects

Thanks to the huge field enhancement of graphene plasmons in GCNWs, lots of other nonlinear effects were investigated, such as four waves mixing [119], second harmonic generation [120], nonlinear plasmon coupling [121], magneto-optical Faraday effect [32,122], etc.

### 4.4. Optical Cloaking

Artificial metamaterials can bend light in almost any manner, and could be used to manipulate electromagnetic waves to achieve optical cloaking based on transformation optics [123,124,125].

**Figure 12:**
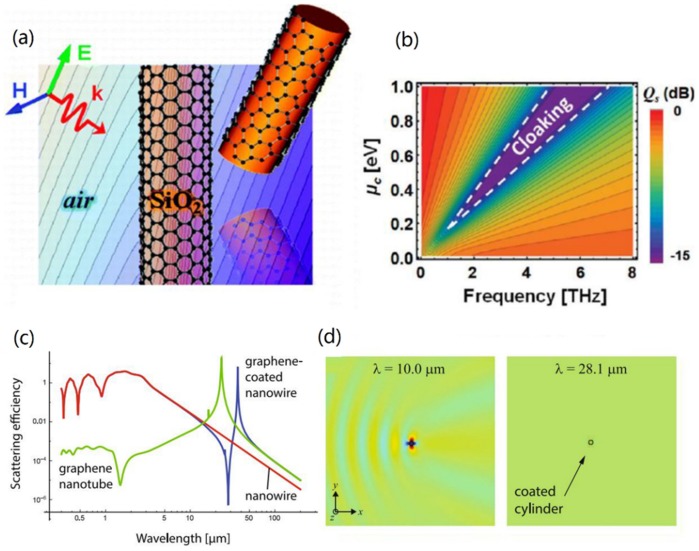
(**a**) Graphene-coated cylindrical object. (**b**) Scattering gain (Q_s_) for the graphene cloak varying chemical potential and the frequency of operation with *τ* = 0.5 ps. Reprinted with permission from reference [126]. Copyright American Chemical Society, 2011. (**c**) Scattering efficiencies (Q_sca_) of three structures. (**d**) Normalized electric field |E/E_0_| for a GCNW with *R* = 0.5 μm illuminated by a transverse-electric (TE)-polarized plane wave at *λ*_0_ = 10.0 μm and 28.1 μm, corresponding to the invisibility window in (**c**). Reprinted with permission from reference [127]. Copyright Springer Nature, 2017.

**Figure 13:**
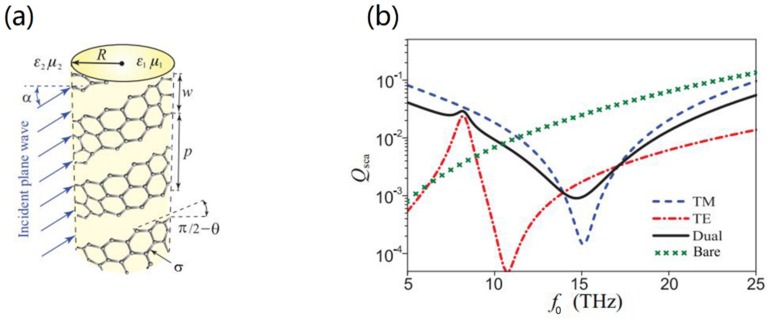
(**a**) Dielectric cylinder coated by helical graphene strips for oblique wave incidence. (**b**) Scattering efficiency versus wave frequency for a GCNW under normal incidence of TE, transverse-magnetic (TM), and dual-polarized waves. Reprinted with permission from reference [129]. Copyright American Physical Society, 2019.

Due to the tunability of surface conductivity of graphene, GCNWs were used to develop tunable invisibility cloaks. For the first time, drastically reduced overall visibility of the scattering object that was conducted via a GCNW in the THz region (see Figure 12a,b) by Chen and Alu [126]. Recently, Naserpour et al. [127] investigated, both theoretically and numerically, a graphene-coated nano-cylinder illuminated by a plane wave. Results showed that the polarization-dependent effect leaded to tunable resonant invisibility that can be achieved via modification of graphene chemical potential. The scattering efficiency and field distributions were shown in Figure 12c,d. Later, invisibility in a trimer of GCNWs was presented by Fesenko et al. [128]. For TM-polarized incident waves, the normalized scattering cross-section spectra of all structures (GCNW, GCNW dimer, GCNM trimer) behaved similarly and exhibited a single invisible region. However, in the case of the TE-polarized incident wave, the normalized scattering cross-section spectra of the GCNW and both dimer and symmetric trimer clusters differed noticeably from each other. The strong coupling between plasmon modes of individual nanowires gave rise to several plasmonic resonances and invisibility regions in the scattering spectra. In 2019, dual-polarized all-angle cloaking was demonstrated [129] by using a helical graphene ribbon coated nanowire, shown in Figure 13a,b. It has been shown that the frequency can be widely tuned with the pitch angle, period, and width of graphene strips, and the most optimal frequency of all-angle cloaking is 13.15 THz.

Furthermore, a modified transformation optics approach was employed to study the plasmonic interactions between two GCNWs [130]. It was found that the interaction between two GCNWs resulted in polarization-independent multi-frequency Fano dips, which showed a broadband red shift of bonding modes and a blue shift of anti-bonding modes when the nanowires approached each other.

### 4.5. Other Applications

Although we have summarized some of the major areas of the GCNW research, the list is far from complete. Lots of other intriguing applications have been demonstrated. For instance, owing to the strong field enhancement of graphene plasmons, Zhu et al. [65] investigated the optical force, which was more than one order of magnitude larger than silver nanowire pairs, in the slot region of the GCNW dimer. Yang et al. [131] studied the optical forces exerted on a graphene-coated dielectric particle by a focused Gaussian beam.

Plasmon-induced-transparency and slow light effect were studied [132] in a GCNW system consisting of two identical-shaped GCNWs placed at either side of the graphene waveguide. Tunable Fano resonance [133] was observed in a system comprised of a point-like emitter near a GCNW. Results showed the Fano line shape of transition rates can be tailored and electrically tuned by varying the distance between emitter and cylinder and by modulating the graphene chemical potential. Recently, a report showed [134] that enhanced electromagnetic energy transfer between the donor and acceptor quantum emitters could be fulfilled by using GCNW surface plasmons.

## 5. Conclusions and Perspective

Although the GCNW is an emerging research field, the achievements in this field have been truly impressive. A lot of promising devices, including waveguides, polarizers, modulators, lasers, nonlinear devices, etc., have been proposed and experimentally demonstrated. However, the applications of GCNWs in other areas where the GCNW can fulfill its potential remain relatively unexplored. For instance, a combination of GCNWs with conventional plasmonic nanostructures. As literature has shown, many GCNW-based subwavelength waveguides have been theoretically investigated, however, there is a lack of experimental work in this area. We also note that the current reports mainly focus on mid- and near-infrared bands, and the application of GCNWs in THz band is relatively less studied. Although graphene plasmons show very strong field localization, due to the high absorption of the graphene layer, the modal propagation length of GCNW-based waveguide is limited. Therefore, further reducing the Ohmic loss is still a huge challenge. Finally, considering the attractive features of strong light–matter interactions, giant field enhancement, and tunability of graphene plasmons, we foresee GCNWs becoming the platform of choice for applications in the fields of the subwavelength photonic devices, super-resolution imaging, enhancing optical force, and nonlinear optics.

## Abbreviations

GCNWs	Graphene-coated nanowires
THz	Terahertz
SPs	Surface plasmons
1/2/3D	One/two/three-dimensional
TM	Transverse-magnetic
TE	Transverse-electric
FWHM	Full width at half maximum
RGO	Reduced graphene oxide
*N*_eff_	Effective mode index
*f*_0_	Frequency
*λ*_0_	Wavelength in vacuum
*μ*_c_	Chemical potential of graphene
*L*_P_	Propagation length
R	Radius of dielectric nanowire
*ε*_d_	Permittivity of dielectric nanowire
